# Miro proteins and their role in mitochondrial transfer in cancer and beyond

**DOI:** 10.3389/fcell.2022.937753

**Published:** 2022-07-25

**Authors:** Zuzana Nahacka, Jaromir Novak, Renata Zobalova, Jiri Neuzil

**Affiliations:** ^1^ Laboratory of Molecular Therapy, Institute of Biotechnology, Czech Academy of Sciences, Prague, Czechia; ^2^ Faculty of Science, Charles University, Prague, Czechia; ^3^ School of Pharmacy and Medical Science, Griffith University, Southport, QLD, Australia

**Keywords:** cancer, mitochondria, intercellular transfer, Miro, respiration, migration, metastasis

## Abstract

Mitochondria are organelles essential for tumor cell proliferation and metastasis. Although their main cellular function, generation of energy in the form of ATP is dispensable for cancer cells, their capability to drive their adaptation to stress originating from tumor microenvironment makes them a plausible therapeutic target. Recent research has revealed that cancer cells with damaged oxidative phosphorylation import healthy (functional) mitochondria from surrounding stromal cells to drive pyrimidine synthesis and cell proliferation. Furthermore, it has been shown that energetically competent mitochondria are fundamental for tumor cell migration, invasion and metastasis. The spatial positioning and transport of mitochondria involves Miro proteins from a subfamily of small GTPases, localized in outer mitochondrial membrane. Miro proteins are involved in the structure of the MICOS complex, connecting outer and inner-mitochondrial membrane; in mitochondria-ER communication; Ca^2+^ metabolism; and in the recycling of damaged organelles *via* mitophagy. The most important role of Miro is regulation of mitochondrial movement and distribution within (and between) cells, acting as an adaptor linking organelles to cytoskeleton-associated motor proteins. In this review, we discuss the function of Miro proteins in various modes of intercellular mitochondrial transfer, emphasizing the structure and dynamics of tunneling nanotubes, the most common transfer modality. We summarize the evidence for and propose possible roles of Miro proteins in nanotube-mediated transfer as well as in cancer cell migration and metastasis, both processes being tightly connected to cytoskeleton-driven mitochondrial movement and positioning.

## Introduction

Mitochondria are cellular powerhouses, generating most of the energy needed for metabolism of cells, as well as orchestrating a number of biochemical reactions from ATP production to amino acids generation or lipid biosynthesis. Except for these pathways, mitochondria regulate cell death, maintain calcium homeostasis and buffer reactive oxygen species (ROS). Mitochondria are highly dynamic organelles that constantly undergo cycles of fusion and fission, and, depending on the cellular type or metabolic status, they differ in size, shape or localization within the cell (Berridge et al., 2016; Furnish and Caino, 2020; Nahacka et al., 2021). They could be efficiently transported to the site of their need, utilizing the system of molecular motors together with tubulin and actin cytoskeleton (MacAskill and Kittler, 2010; Kittler, 2015; Eberhardt et al., 2020; Grossmann et al., 2020). Furthermore, mitochondria communicate with other organelles, often forming physical bridges with the endoplasmic reticulum (ER) or the nucleus (Modi et al., 2019; Desai et al., 2020).

For a long time it was thought that, mitochondria are not essential for cancer cells due to the dependence of tumor energy production on glycolysis (Warburg, 1956; Vander Heiden et al., 2009; Porporato et al., 2018; Vaupel et al., 2019; Pavlova et al., 2022). This misinterpretation has been corrected over the last decades, such that now we know that while mitochondrial ATP production is dispensable for tumorigenesis, mitochondria-linked pyrimidine biosynthesis is important to drive proliferation of cancer cells (Bajzikova et al., 2019; Boukalova et al., 2020). Our research as well as research of others has shown that cancer cells lacking mtDNA are able to form tumors only if they acquire respiration-competent mitochondria from neighboring cells of tumor stroma (Tan et al., 2015; Berridge et al., 2016; Dong et al., 2017; Bajzikova et al., 2019). Additionally, emerging evidence shows the importance of mitochondria in the process of tumor cell migration, invasion of distant tissues and formation of metastases (Jiang et al., 2012; Caino et al., 2016; Furnish and Caino, 2020; Ghosh et al., 2022).

In this short review, we will briefly discuss the role of Miro proteins in cancer and tumor microenvironment, especially in intercellular mitochondrial transfer from donor stromal cells to cancer cells focusing on tunneling nanotubes that are important for mitochondrial transfer between cells; the step that is necessary to initiate tumor cell proliferation and cancer progression during aberrant respiration. Additionally, we will also discuss the role of these proteins in spatial distribution of mitochondria and mitochondrial network, plus their role in the process of tumor cell invasion and metastasis. The comprehensive review of structure and functions of Miro1 and Miro2 is a focus of previous review articles by others and us (Nahacka et al., 2021). For more information, see Table 1.

**TABLE 1:** Summary of review articles focusing on particular aspects of intercellular mitochondrial transfer, tunneling nanotubes and various functions of Miro proteins in physiological conditions and in different pathologies.

Topic	Author	Note
Different modes of mitochondrial transfer	Sinha et al. (2016)	Intercellular mitochondrial transfer in organs and cells
	Torralba et al. (2016)	The physiological relevance and therapeutic application for treating mitochondrial-related diseases
	Valenti et al. (2021)	Intercellular mitochondrial transfer in physiological and pathological conditions
	Vona et al. (2021)	Mitochondrial dynamics in cancer and potential therapeutic approach
	Zampieri et al. (2021)	Different modes of mitochondrial intercellular transfer in cancer
Structure and formation of TNTs	Dagar et al. (2021)	Molecular mechanisms regulating TNTs formation
	Drab et al. (2019)	Molecular mechanism and biophysics of TNTs formation
	Ljubojevic et al. (2021)	Detail summary of actin-related molecules and transfer mechanisms
	Sahu et al. (2018)	Molecular pathways involved in TNTs formation
Comprehensive TNT-focused review	Cordero Cervantes and Zurzolo (2021)	Comprehensive review of various aspects of TNTs (methodology, signaling, cargos, structure)
	Pinto et al. (2020)	TNT-mediated transfer in cell lines and in tissues
	Qin et al. (2021)	Signaling and formation of TNTs, and actin-mediated transfer of mitochondria
	Vignais et al. (2017)	General review regarding mitochondrial transfer
TNTs in mitochondrial transfer in cancer	Allegra et al. (2022)	TNTs in leukemia, possible therapeutic approach
	Berridge and Neuzil (2017)	Mitochondrial transfer *in vitro* and in mouse models
	Hekmatshoar et al. (2018)	Role of mitochondrial transfer in metabolic plasticity of cancer cells and resistance to therapy
	Pinto et al. (2020)	TNT-mediated transfer in cell lines and in tissues
	Roehlecke and Schmidt (2020)	TNT-mediated transfer of various cargo and its impact on tumor progression and therapy resistance
	Sahinbegovic et al. (2020)	Mitochondrial transfer in solid tumors and hematological malignancies
TNTs role in different pathologies	Khattar et al. (2022)	TNTs in physiological and pathological conditions in the brain
	Mittal et al. (2019)	Comprehensive summary of TNTs in various cell types and the use of inhibitors of TNTs and of mitochondrial transfer
	Tiwari et al. (2021)	TNTs in various deseases
Miro and other GTPases in TNT formation	Qin et al. (2021)	Signaling and formation of TNTs, and actin-mediated transfer of mitochondria
	Raghavan et al. (2021)	Role of mitochondrial transfer via TNTs in various pathologies. Various roles of Rho GTPases related to TNTs
Miro and its role in various pathologies	Brunelli et al. (2020)	Role of Miro proteins in Parkinson desease
	Kay et al. (2018)	The role of mitochondrial transport and Miro proteins in different neurodegenerative deseases
	Kruppa et al. (2018)	Role of Miro proteins and cytoskeletal motors in mitochondria homeostasis
	Panchal and Tiwari (2021)	Miro proteins and their role in the pathogenesis of various neurodegenerative deseases
	Shanmughapriya et al. (2020)	Miro, molecular motors, adaptors and transport of mitochondria in CNS
Comprehensive review focused on structure and functions of Miro proteins	Eberhardt et al. (2020)	Comprehensive review about Miro proteins
	Nahacka et al. (2021)	Various aspects of Miro proteins in cell physiology and mitochondrial movement
	Ming Yang et al. (2020)	Comprehensive review of ER and mitochondria contact sites and signaling molecules involved in their formation
	Zinsmaier (2021)	Comprehensive review about Miro proteins
Miro proteins in cell migration	Furnish and Caino (2020)	Role of intracellular mitochondrial localization in cancer cell invasion
Metabolism of cancer cells	Boukalova et al. (2020)	Comprehensive review concerning DHODH role in cancer cells and potential therapeutic intervences
	Pavlova et al. (2022)	Review on metabolism and regulation of gene and protein expression in tumor microenviroment
	Porporato et al. (2018)	Review on role of mitochondria in all steps of oncogenesis

## Miro proteins, their structure and function

Miro1 and Miro2 proteins (encoded by *RHOT1* and *RHOT2* genes, respectively) are main adaptors transporting mitochondria inside cells (Saotome et al., 2008; MacAskill et al., 2009a; Eberhardt et al., 2020). Miro proteins are evolutionary conserved throughout the eukaryotes with one homolog in yeasts (Gem1p) and lower metazoans (in *D.melanogaster* dMiro) and two homologs Miro1 and Miro2 in mammals (Frederick et al., 2004; Guo et al., 2005; Li X. et al., 2015; Beljan et al., 2020). These proteins classified as a specific subfamily of small GTPases comprise 620 amino acid residues and share 60% of sequence homology (Wennerberg and Der, 2004; Fransson et al., 2006; Reis et al., 2009). They are type II trans-membrane proteins embedded in the outer mitochondrial membrane consisting of the C-terminal trans-membrane domain and two canonical Ca^2+^ binding EF hand motifs flanked by two GTPase domains (the N-GTPase and the C-GTPase) (Fransson et al., 2006; MacAskill et al., 2009a; Reis et al., 2009; MacAskill and Kittler, 2010; Klosowiak et al., 2013). The C-terminal GTPase domain forms together with two EF-hand motifs, a rigid structure called MiroS (Klosowiak et al., 2013). While EF-hand domains feature calcium binding function, the two GTPase domains are both structurally and functionally distinct. It is thought that the more evolutionary conserved N-GTPase domain is of a greater importance for the function of the protein than the C-GTPase domain, supported by mutation studies showing a more severe impact on the phenotype in the case of the N-terminal domain mutation (Fransson et al., 2006; Reis et al., 2009; Smith et al., 2020). Except for nucleotide binding and GTP hydrolase activity of both domains, it was proposed that the C-GTPase domain possesses also nucleoside triphosphatase (NTPase) function (Peters et al., 2018; Smith et al., 2020). Miro exists in protein complexes, its interacting partners are mostly dimers or multimers (TRAK1/2, Kinesin-1, Mfn1/2), but the exact stoichiometry of Miro complexes needs to be elucidated. It was shown by crystallization studies that the N-GTPase domain has potential binding interfaces, which mediate homo-dimerization in the crystal (Smith et al., 2020).

Miro1 and Miro2 proteins are ubiquitously expressed with high level of Miro1 in the heart and in skeletal muscles and high level of Miro2 in the heart, liver, skeletal muscle, kidney and pancreas (UniProt Consortium, 2019). Despite their homology, they are able to compensate each other only to limited degree. During embryogenesis, both proteins are needed in different stages of development (Lopez-Domenech et al., 2016; Lopez-Domenech et al., 2018). Miro1 knock-out (Miro1 KO) is embryonically lethal while Miro2 knock-out (Miro2 KO) mice were found to develop normally until adulthood (Lopez-Domenech et al., 2016). Although Miro1 was able to compensate for the loss of Miro2, this did not happen in the opposite situation (Lopez-Domenech et al., 2016). Miro1 deletion led to disrupted mitochondrial trafficking and aberrant distribution of the organelles in dendritic neurons *in vitro* and *in vivo*, resulting in neurodegeneration.

### Mitochondrial trafficking

It is known that Miro1 is a key regulator of mitochondrial trafficking not only in neurons but also in other cell types (Yamaoka et al., 2011; Kittler, 2015; Stephen et al., 2015; Lopez-Domenech et al., 2016; Lopez-Domenech et al., 2018; Kalinski et al., 2019; Eberhardt et al., 2020; Nahacka et al., 2021). Both proteins were found to bind protein complexes involved in tubulin (kinesin/dynein and TRAK1/2 complex) and actin (myosin XIX or Myo19) movement, but their participation in these processes is quite different (Brickley et al., 2005; Fransson et al., 2006; Glater et al., 2006; MacAskill et al., 2009a; Lopez-Domenech et al., 2018; Oeding et al., 2018; Bocanegra et al., 2020). As mentioned, Miro1 has a dominant function in mitochondrial localization and trafficking at long-range transport utilized by tubulin cytoskeleton (Lopez-Domenech et al., 2018). Bidirectional trafficking of mitochondria along microtubules is considered the dominant type of mitochondrial movement in metazoans (Schwarz, 2013). When observing anterograde and retrograde mitochondrial trafficking in hippocampal neurons or MEF cells *in vitro*, Miro2 KO did not have a significant effect while Miro1 KO decreased the extent of mitochondrial movement (Lopez-Domenech et al., 2018). Overexpression of Miro2 was not able to fully rescue the Miro1 KO phenotype (Lopez-Domenech et al., 2016; Lopez-Domenech et al., 2018). Even though ablation of the *RHOT2* gene did not affect the localization of mitochondria in MEF cells, Miro2 protein was found to be partially involved in this process, since Miro1 and Miro2 double knock-out (DKO) accentuated phenotype (Lopez-Domenech et al., 2018). Still Miro2 cannot fully compensate for the loss of Miro1 and its role seems to be somewhat redundant in the presence of Miro1. Interestingly, both Miro proteins regulate actin-based movement with more dominant role of Miro2 in the short-range transport (Lopez-Domenech et al., 2018). Actin filaments were shown to be involved not only in short-range transport of mitochondria but also in dynamic processes such as fusion and fission or during mitosis in the proper segregation of mitochondria to daughter cells (Quintero et al., 2009; Korobova et al., 2014). The prevalent role of Miro2 in actin movement was reflected also in unequal segregation of mitochondria during mitosis (Lopez-Domenech et al., 2018). Miro proteins not only bind Myo19, the molecular motor moving mitochondria along actin filaments, but also stabilize it and protect it from degradation (Lopez-Domenech et al., 2016; Oeding et al., 2018). How Miro1 and Miro2 decide between different protein complexes when binding TRAK1/2 or Myo19 is still a question that needs to be answered. Interestingly, even in the absence of both Miro proteins a movement of mitochondria in a TRAK/kinesin-dependent manner could be detected (Lopez-Domenech et al., 2018). Most probably there are still unknown protein(s) anchoring mitochondria to tubulin at least for anterograde movement along tubulin fibers that is presumably also responsive to calcium regulation. Mitofusins Mfn1/2 could possibly play this role, since they were found to be bound in a complex with TRAK/Miro, and Mfn2 was shown to be necessary for axonal transport of mitochondria in *D. melanogaster* (Misko et al., 2010; Lee C. A. et al., 2018). Furthermore, in a recent study on *C. elegans* metaxins (*MTX-1* and *MTX-2*), components of the protein import complex across the outer mitochondrial membrane (OMM), were identified as proteins capable to bind mitochondria to kinesin or dynein motors and bypass (but not fully recover) Miro failure (Zhao et al., 2021). Additionally, Myo19 was shown to be involved in Miro-independent interaction with mitochondria *via* its C-terminal membrane-association domain (MyMOMA) (Bocanegra et al., 2020).

Two main reservoirs of Ca^2+^ ions in cells are the endoplasmic reticulum and the mitochondria (Chang et al., 2011). Calcium is a general regulator of mitochondrial processes from trafficking to mitophagy, also affecting mitochondria-ER contact sites (MERCS) and processes such as apoptosis and mitochondrial bioenergetics (Wan et al., 1989; Saotome et al., 2008; MacAskill et al., 2009b; Stephen et al., 2015; Lee et al., 2016; Safiulina et al., 2019a; Safiulina et al., 2019b; Modi et al., 2019; Romero-Garcia and Prado-Garcia, 2019; Patergnani et al., 2020). Miro proteins have two EF-hand Ca^2+^-binding motifs. When the cytoplasmic concentration of Ca^2+^ increases, Miro binds calcium and changes its conformation that results in the release of the adaptor/motor complex from the cytoskeleton (Saotome et al., 2008; Klosowiak et al., 2013). Additionally, not only cytoplasmic but also mitochondrial calcium level probably impact on the frequency of mitochondria movement (Chang et al., 2011).

### Miro as interacting partner of mitochondrial contact-site and cristae organizing system complex

Miro proteins are tethered in the OMM, and one can presume that their deletion will affect mitochondrial morphology and architecture. Both proteins were shown to be a part of the mitochondrial contact-site and cristae organizing system (MICOS) complex that connects OMM and the inner mitochondrial membrane (IMM) and that is important for the cristae architecture maintenance (Hoppins et al., 2011; Tsai et al., 2017; Modi et al., 2019; Yang M. et al., 2020; Stephan et al., 2020). Interestingly both Miro proteins equally cooperate in the regulation of mitochondrial morphology and, despite the changes in the shape of mitochondria and altered cristae organization; ablation of both genes does not affect the maximum respiratory capacity of the electron transport system *in vitro* (Lopez-Domenech et al., 2016; Modi et al., 2019)*.* One of the reasons of this phenomenon could be that Miro DKO did not change the expression of components of the MICOS complex but rather altered its membrane distribution (Modi et al., 2019). More experiments are needed to see how Miro DKO affects the bioenergetics of cells and whether alteration of cristae architecture results in changes of the respiratory protein complexes composition.

In addition to movement of mitochondria along tubulin and actin cytoskeleton, Miro proteins were found to have a function in the process of mitophagy and regulation of MERCS (Kay et al., 2018; Eberhardt et al., 2020). Interestingly here they seem to be equally important and are able to mutually compensate each other suggesting that functional differences in mitochondrial trafficking would be more a question of different affinity to bind to molecular motors or to participate in different protein complexes involved in transport of mitochondria (Safiulina et al., 2019a; Safiulina et al., 2019b; Modi et al., 2019).

### Mitophagy regulation

Mitophagy is a specific type of autophagy occurring in the case of energetic disbalance and defective mitochondria (Kittler, 2015; Pickles et al., 2018). One of the types of mitophagy was shown to be regulated by PINK1 kinase that activates Parkin, a ubiquitin ligase that ubiquitinates several downstream targets located in the OMM, including Miro proteins (Greene et al., 2012; Kane et al., 2014; Lazarou et al., 2015). These targets comprise, for example, a group of import receptors and ion channels such as TOM20, VDAC1 or VDAC3, and Mfn1 and Mfn2 proteins involved in the process of fusion and fission to block the fusion of defective mitochondria and Miro proteins (Geisler et al., 2010; Wang X. et al., 2011; Sun et al., 2012; Bingol et al., 2014; Birsa et al., 2014; Choubey et al., 2014). Parkin further facilitates K48 and K63 ubiquitination of substrates and recruits the autophagy machinery and complexes of lysosomal degradation (Olzmann et al., 2007; Moore et al., 2008; Richard et al., 2020; Park et al., 2021). Degradation of Miro by Parkin is a prerequisite for the arrest of mitochondrial movement, so that clearance of mitochondria can properly proceed (Wang X. et al., 2011; Birsa et al., 2014).

Miro proteins were found to be more than mere downstream targets for Parkin ubiquitination, themselves being active regulators of this process. Miro1 directly interacts with PINK1, whereby enhancing the catalytic activity of Parkin (Wang X. et al., 2011; Park et al., 2017). Moreover, both Miro proteins are required for proper Parkin translocation to OMM and for initiation of mitophagy (Safiulina et al., 2019a; Safiulina et al., 2019b). This process occurs independently of PINK1 activity or their own degradation and is regulated by calcium-binding ability of EF-hand domains of Miro proteins (Safiulina et al., 2019a; Safiulina et al., 2019b). Higher concentrations of calcium not only arrest mitochondrial movement by decreasing the affinity of Miro for its binding partners involved in mitochondrial transport but it also serves as a switch, recruiting (by Miro) cytosolic Parkin to mitochondria; priming mitochondria for the process of mitophagy (Yi et al., 2004; Saotome et al., 2008; Safiulina et al., 2019a; Safiulina et al., 2019b).

Dysregulation of mitophagy (and calcium signaling) is linked to many pathologies, starting with neurological diseases such as Parkinson disease (PD), heart failure, metabolic disorders, aging and cancer (Lahiri and Klionsky, 2017; Kay et al., 2018; Pickles et al., 2018; Cao et al., 2019; Kesharwani et al., 2019; Berenguer-Escuder et al., 2020; Brunelli et al., 2020; Eberhardt et al., 2020; Imai, 2020; Denisenko et al., 2021; Nahacka et al., 2021; Zhang et al., 2021; Babula and Krizanova, 2022; Diao and Gustafsson, 2022; Lee et al., 2022; Shan et al., 2022). Miro proteins are important regulators of the process of mitophagy, firstly prior to mitochondrial damage where they serve as calcium sensors, translocating Parkin to mitochondria, and then as Parkin downstream targets where ubiquitination of Miro proteins arrests mitochondrial trafficking needed for the organelle removal (Nahacka et al., 2021).

### Involvement of Miro in mitochondria-ER contact sites

Connections between the endoplasmic reticulum and mitochondria (MERCS) have been described for single cell organisms as well as for metazoans (Vance, 1990; Frederick et al., 2004). MERCS are involved in many cellular functions. Both organelles serve as a reservoir of calcium; therefore, MERCS are involved in the regulation of calcium signaling (Rizzuto et al., 1998). Linked to this MERCS serve as a signaling hub for lipid metabolism, insulin signaling, mitochondrial fission, autophagy and apoptosis (Vance, 1990; Simmen et al., 2005; Friedman et al., 2011; Hamasaki et al., 2013; Tubbs et al., 2014; Sala-Vila et al., 2016; Rieusset, 2017). Gem1p, a homolog of Miro, was found to be a binding partner of ERMES (ER-mitochondria encounter structures) complex in yeasts (Frederick et al., 2004; Kornmann et al., 2011; Koshiba et al., 2011). A deletion study showed that the role of Gem1p is more in the regulation of ERMES than in the architecture of these structures (Kornmann et al., 2011). Interestingly calcium binding EF domains and N-GTPase domain were found necessary for the association of this protein with ERMES. Another example of Miro homolog with a function in mitochondria-ER contact sites (MERCS) was found in *D. melanogaster* (Lee et al., 2016; Lee K. S. et al., 2018)*.* An important study showed that localization and interaction of dMiro with calcium transporters in MERCS and thus calcium homeostasis is dependent on phosphorylation of Miro by Polo kinase (Lee et al., 2016).

Interaction of Miro with calcium uniporters was shown also for humans (Niescier et al., 2013; Niescier et al., 2018). Calcium levels in *Drosophila* and the dMiro status were found to be important for proper brain development regardless of gain-of-function or loss-of-function mutation of dMiro (Lee et al., 2016; Lee K. S. et al., 2018). In both cases, mutations led to impaired calcium signaling that resulted either in activation of mitochondria-induced apoptosis or ATP depletion and cell growth inhibition, respectively. Polo kinase was shown to be important for brain development in not only lower organisms such as *Drosophila*, but its impaired function could also be found in humans in neurological diseases (e.g., PD) (Mbefo et al., 2010; Elfarrash et al., 2021; Weston et al., 2021).

Interestingly deregulated activity of this kinase was shown to be associated also with cancer (Naik et al., 2011; Craig et al., 2014). Other genes participating in the regulation of MERCS were shown to be related to PD in humans, such as PINK1, Parkin, leucine-rich repeat kinase 2 (LRRK2), α-synuclein and Miro. All of these genes are involved in regulation of mitochondrial quality control and mitophagy, processes connected by calcium signaling. Several studies showed a connection between *RHOT1* gene mutations and disruption of Miro1 function in MERCS in PD patients (Berenguer-Escuder et al., 2019; Grossmann et al., 2019; Berenguer-Escuder et al., 2020; Grossmann et al., 2020). In all cases regardless of the type of mutation of *RHOT1* gene, this resulted in the decrease of MERCS and eventually deregulated calcium signaling due to diminished connections between two organelles serving as reservoirs of calcium ions. Interestingly, gene-based association clustering methods revealed correlation between *RHOT2* gene mutations and PD (Saeed, 2018). Moreover, equal contribution of Miro1 and Miro2 in regulation of MERCS was observed in MEFs isolated from knock-out mice (Modi et al., 2019). Re-expression of either Miro1 or Miro2 was able to rescue the WT phenotype, recover the extent of contacts between ER and mitochondria, and restore calcium signaling.

## Miro proteins and different modes of mitochondrial transfer

The ability of cells to communicate *via* exchange of cellular components such as mitochondria is crucial for maintaining homeostasis in multicellular organisms. It has been shown that it also plays an important role in pathological states such as cancer, pulmonary disease, cardiomyopathy and brain damage (Islam et al., 2012; Tan et al., 2015; Hayakawa et al., 2016; Moschoi et al., 2016; Sinha et al., 2016; Torralba et al., 2016; Dong et al., 2017; Paliwal et al., 2018; Nicolas-Avila et al., 2020; Valenti et al., 2021; Vona et al., 2021; Zampieri et al., 2021). Mitochondrial transfer is triggered in response to various signals, most likely originating in the recipient cell; however, the molecular signals that initiate this “cross-talk” are still not fully clear.

There are several mechanisms of mitochondrial movement between cells, i.e., from the donor to the recipient cell. Tunneling nanotubes, actin-based cytoplasmic bridges, are generally perceived as the prevalent cellular structure that mediates intercellular mitochondrial transfer (Rustom et al., 2004; Gerdes and Carvalho, 2008; Vignais et al., 2017; Sahu et al., 2018; Drab et al., 2019; Cordero Cervantes and Zurzolo, 2021; Ljubojevic et al., 2021). Other proposed mechanisms include extracellular vesicles (EVs), which are secreted into the extracellular milieu by most cell types. EVs, ranging in size from 40 to 1,000 nm, can be divided into microvesicles, exosomes and apoptotic bodies, depending on their size, origin and molecular composition (Mittelbrunn and Sanchez-Madrid, 2012; Amari and Germain, 2021; Lazar and Goldfinger, 2021; Levoux et al., 2021). Interestingly, it was recently shown that EVs can be transported over longer distances as in inter-organ transport of mitochondria between energetically stressed adipocytes and cardiomyocytes (Crewe et al., 2021). Furthermore, the authors showed formation of mitochondria-derived vesicles (MDVs) that are packed into EVs and transport mitochondria to other cells (Crewe et al., 2021). It has been reported that Miro proteins, as part of outer mitochondrial membrane, are involved in the formation of MDVs (Konig et al., 2021). These particles are formed by budding of mitochondrial membranes and later fuse with either lysosomes or late endosomes (Soubannier et al., 2012). MDV forming was originally described for bacteria and serves as quality control of mitochondria (Soubannier et al., 2012; Pickles et al., 2018). Moreover, MDVs utilize communication between mitochondria and other organelles within the cells, especially with peroxisomes (Sugiura et al., 2017).

Other proposed mechanisms allowing for movement of mitochondria between cells include gap junctions and cell fusion. Gap junctions are clusters of intercellular channels that directly connect the cytoplasm of two adjacent cells allowing for direct diffusion of ions, small molecules and electrical pulses (Goodenough et al., 1996; Paliwal et al., 2018; Qin et al., 2021; Zampieri et al., 2021). Cell fusion is a process in which 2 cells fuse *via* their membranes, sharing organelles and cytosolic components, while nuclei remain intact (Aguilar et al., 2013; Valenti et al., 2021; Zampieri et al., 2021). Finally, mitochondrial extrusion is another proposed mechanism governing transfer of mitochondria between cells (Sinha et al., 2016; Torralba et al., 2016).

Notwithstanding the various modes of intercellular transfer of mitochondria, movement of the organelles between cells occurs in many cases *via* tunneling nanotubes, which is central for this review.

## Tunneling nanotubes

### Structure and formation of TNTs

TNTs were first described as several micrometers long actin-containing membrane connections formed between PC12 cells *in vitro*, mediating transfer of vesicles and organelle (Rustom et al., 2004). Since this initial observation, TNTs have been observed in a number of both cancer and non-cancerous cell types, both *in vitro* and *in vivo* (Islam et al., 2012; Jiang et al., 2016; Berridge and Neuzil, 2017; Hekmatshoar et al., 2018; Mittal et al., 2019; Pinto et al., 2020; Sahinbegovic et al., 2020; Tiwari et al., 2021; Allegra et al., 2022; Khattar et al., 2022). Although capable to move many different cargos (miRNA, lysosomes, liposomes, Golgi vesicles, calcium, etc.) across connections lasting for minutes to hours, most common cargo transferred *via* TNTs are mitochondria. Under physiological conditions, transfer of mitochondria from multipotent cells may serve as a mechanism of “rejuvenation” of the recipient cells, e.g., shifting them to more progenitor-like phenotype (Acquistapace et al., 2011). Inversely, transport of mitochondria from differentiated to multipotent cells can enhance their proliferation or trigger their differentiation into another cell type (Plotnikov et al., 2010; Vallabhaneni et al., 2012). Moreover, transfer of mitochondria was also demonstrated in differentiated cells between cardiomyocytes and cardiofibroblasts with TNTs being detected both *in vitro* and in the tissue (He et al., 2011).

In cancer, the formation of TNTs and intercellular exchange of mitochondria serves for maintaining the metabolic homeostasis of cancer cells and could be the reason of increased malignancy of cancer cells and cancer drug resistance (Moschoi et al., 2016; Rustom, 2016; Marlein et al., 2017; Hekmatshoar et al., 2018; Sahu et al., 2018; Marlein et al., 2019; Raghavan et al., 2021). One of the first pieces of evidences of TNT formation in malignant tissues comes from solid tumors isolated from patients with lung adenocarcinoma and pleural mesothelioma followed by similar observations in patient-derived ovarian and pancreatic cancer tissues (Lou et al., 2012b; Thayanithy et al., 2014; Desir et al., 2018). Later on, mitochondrial transfer *via* TNTs was observed in glioblastomas, multiple myelomas or in an *in vitro* model of breast cancer, laryngeal cancer, bladder cancer and others (Pasquier et al., 2013; Antanaviciute et al., 2014; Osswald et al., 2015; Lu et al., 2017; Marlein et al., 2019; Pinto et al., 2020; Salaud et al., 2020; D’Aloia et al., 2021; Pinto et al., 2021). Similarly, cancer cells have been shown to gain malignancy or enhanced resistance to drug treatment, hypoxia or apoptosis *via* TNTs (Pasquier et al., 2013; Ady et al., 2014; Wang and Gerdes, 2015; Desir et al., 2016; Rustom, 2016; Desir et al., 2018; Hekmatshoar et al., 2018; Sahu et al., 2018; Raghavan et al., 2021; Kato et al., 2022). Moreover, TNTs were identified in hematological malignancies, where inhibition of their formation improved the survival of diseased mice *in vivo* and enhanced chemosensitivity *in vitro* (Polak et al., 2015; Wang et al., 2018; Marlein et al., 2019). Interestingly, recent work illustrates that cancer cells transfer mitochondria *via* TNTs from lymphocytes, depleting immune cells on top of gaining metabolic advantage (Saha et al., 2022).

TNTs are classified as cell protrusions that 1) connect two (or more) cells; 2) do not touch the substrate; and 3) contain F-actin (Dupont et al., 2018; Roehlecke and Schmidt, 2020; Shanmughapriya et al., 2020). TNTs can connect cells of similar or different types, based on this they are referred to as homo- or hetero-TNTs (Dubois et al., 2020). Reported length of TNTs varies from tens to hundreds of micrometers and width from fifty to fifteen hundred nanometers (Ady et al., 2014; Austefjord et al., 2014; Dubois et al., 2020). There are two groups of TNTs, the first category includes thicker tubes (>700 nm) containing both tubulin and F-actin, whereas the second thinner tubes are composed of actin only (Sahu et al., 2018). This simple taxonomy was however recently challenged by the observation that thick TNTs formed by thin individual TNTs (or iTNTs) are bundled together by cadherin or spiraled up into thick TNTs (Sartori-Rupp et al., 2019; Franchi et al., 2020). Presence of intermediate filaments, the third main frequent cytoskeletal component, is much less understood as was reported in several studies, and most probably provides mechanical support (Ady et al., 2014; Resnik et al., 2018; Dubois et al., 2020; Latario et al., 2020).

There are several proposed mechanisms of TNT formation. They can start forming as filopodia or similar cellular protrusion from one of the cells, which will be connected with the other cell or they can form as residues of cell membranes after migration of cells away from each other (Sahu et al., 2018; Dubois et al., 2020; Roehlecke and Schmidt, 2020). As TNTs improve cell fitness under unfavorable conditions such as inflammatory environment, hypoxia or metabolic stress, their induction is connected to stress-related (p53, MAP kinase) and pro-survival (EGFR, Akt, PI3K or mTOR) signaling pathways promoting remodeling of the cell cytoskeleton and plasma membrane (Lou et al., 2012b; Ahmad et al., 2014; Desir et al., 2016; Sahu et al., 2018). Generally, there are three triggers resulting in TNT formation, often acting together: chemical or physical stressors (pH, hypoxia), inflammatory conditions (infection, toxins—LPS, or cytokines) and metabolic stress (starvation). Various inflammatory conditions have been demonstrated to induce TNTs such as LPS in cardiomyocytes or TNFα and IL-13 in epithelial cells. In both cases treatment induces the formation of more TNTs by the surrounding MSCs, which donate mitochondria (Ahmad et al., 2014; Yang Y. et al., 2020). In another study, RAGE receptors and subsequent MAP kinase signaling were shown to be involved in TNT formation in response to inflammatory conditions (Sun et al., 2012; Ranzinger et al., 2014). RAGE receptors are of exceptional interest as they are involved in both inflammation and oxidative stress responses, and they have been hypothesized to contribute to tissue homeostasis *via* TNT formation under various stress conditions (Rustom, 2016). Hypoxia, oxidative stress in the form of hydrogen peroxide and metabolic stress induced by serum depletion or mitochondrial damage induce or enhance TNT formation, illustrating that they present most likely a general mechanism utilized by distinct cell types in response to the various manifestations of mitochondrial malfunction (Wang Y. et al., 2011; Lou et al., 2012a; Lou et al., 2012b; Ahmad et al., 2014; Liu et al., 2014; Thayanithy et al., 2014; Desir et al., 2016; Jiang et al., 2016; Kretschmer et al., 2019; Yang Y. et al., 2020). Details of signaling molecules and pathways that bridge cellular stress and formation of TNTs are far from being elucidated, and our understanding is now rather contradictory. P53, a central hub of stress-related signaling pathways, was first described as essential for TNT formation, but later reported to be dispensable for this process (Wang Y. et al., 2011; Andresen et al., 2013; Zhang and Zhang, 2015). Other molecules reported to be involved in TNT formation include ROCK, PAK, MAP/ERK, PI3K, mTOR, and, thoroughly reviewed elsewhere (see Table 1).

Downstream from the above mentioned signaling pathways are proteins involved in TNT formation *via* interaction with the actin cytoskeleton or cell membrane. One such protein identified in several human and mouse cells is M-Sec (TNFAIP2), that regulates actin polymerization, filopodia formation and exocyst complex recruitment *via* interaction with small GTPases from the Rho (Cdc42) and Ral-like families (RalA) (Hase et al., 2009; Kimura et al., 2016; Lotfi et al., 2020; Barutta et al., 2021). Other proteins are the Arp2/3 complex branching actin, LST1 recruiting exocyst, RalA and actin cross-linking protein filamin or the unconventional motor protein Myo10 (Gousset et al., 2013; Schiller et al., 2013; Hanna et al., 2017; Tasca et al., 2017; Latario et al., 2020). Also, cell adhesion molecules and receptors (FAK, ICAM-1, or CD38) are frequently reported to be involved in TNT formation and maintenance or to correlate with mitochondrial transfer *via* TNTs (Sáenz-de-Santa-María et al., 2019; Wang et al., 2018; Marlein et al., 2019).

### Mechanism of mitochondrial transfer *via* TNTs and the role of Miro proteins

By definition, all TNTs contain actin cytoskeleton and, in many cases, microtubules, both of these structures possibly acting as “tracks” for movement of mitochondria. Microtubule-associated motors dynein or kinesin mediate the movement of mitochondria towards minus or plus ends of microtubules over long distances, in contrast to short-range actin-mediated transport (MacAskill and Kittler, 2010). Miro proteins provide tethering of mitochondria to cytoskeletal motor proteins and serve as regulators of transport of the organelles, based on their calcium-dependent activity (Eberhardt et al., 2020; Nahacka et al., 2021). Essential role of Miro in intercellular transfer of mitochondria has been demonstrated by many studies reporting a correlation between Miro expression and mitochondria exchange with TNTs being the conduits for the transport (Ahmad et al., 2014; Babenko et al., 2018; English et al., 2020; Tseng et al., 2021; Wang et al., 2021). Except for one study, presence of Miro was found essential for the transfer/movement of mitochondria along TNTs rather than for their formation (Babenko et al., 2018; Raghavan et al., 2021). Although direct evidence connecting Miro to a particular alteration in TNT’s dynamic or structure is still missing, the process of TNTs formation *via* cytoskeleton and membrane remodeling is energetically demanding, with positioning of mitochondria (involving Miro) likely playing a role. It is still questionable, whether Miro proteins are more important for transport involving microtubules or actin structures as tracks for movement of mitochondria. Notwithstanding this unresolved question, given the notion that mitochondria move between cells over relatively long distances, it is more probable that Miro proteins are involved in tubulin-based transport. However, rather infrequent and contradictory reports regarding the importance of microtubules for TNT-mediated transfer between cells and reports of presence of mitochondria in TNTs lacking microtubules give rise to more questions that need to be answered (Luchetti et al., 2012; Wittig et al., 2012; Hanna et al., 2017; Sartori-Rupp et al., 2019).

**FIGURE 1:**
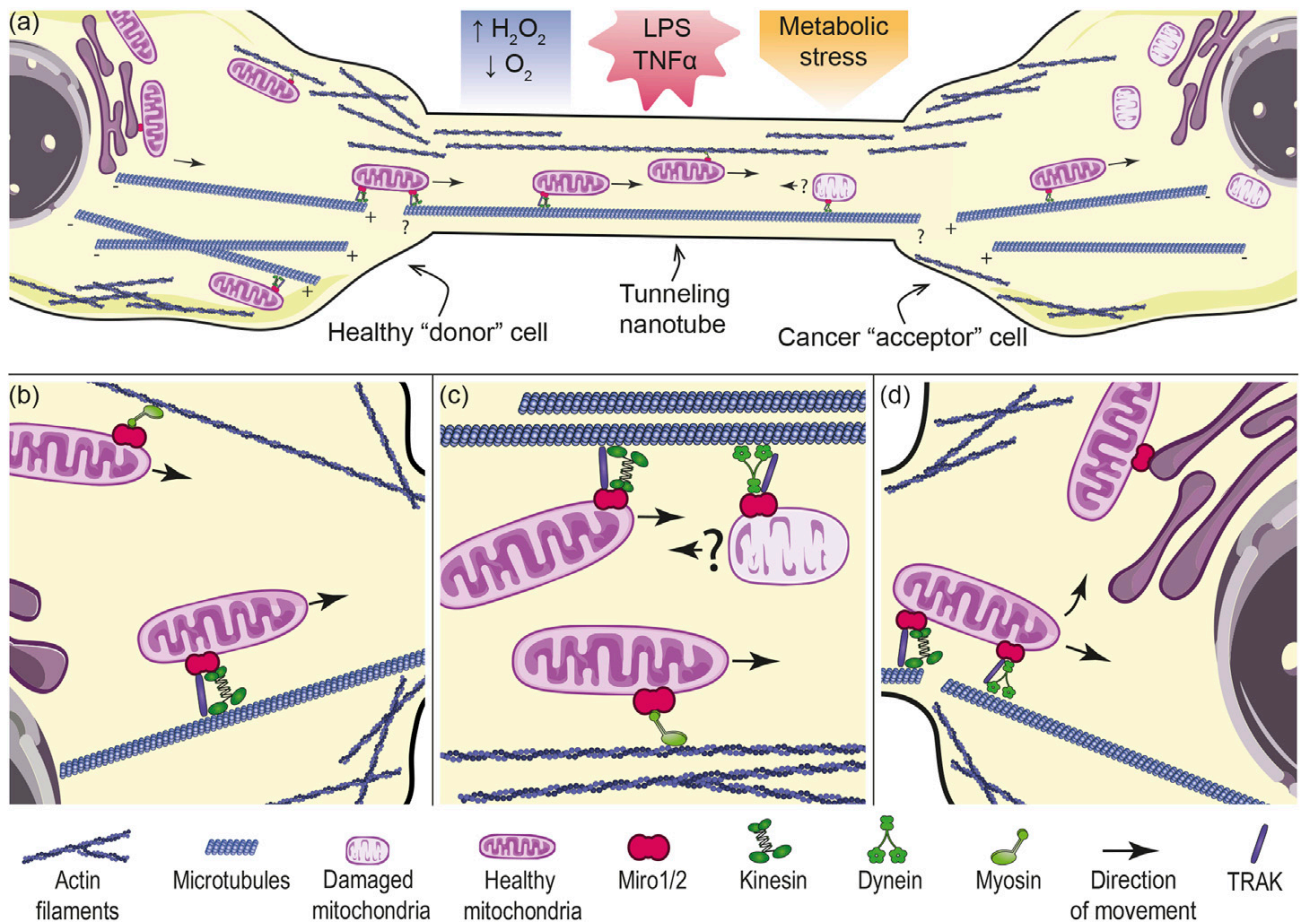
Mechanism of mitochondrial transport between two cells connected via TNTs. **(A)** Tunneling nanotube (TNT) formation between donor and acceptor cell could be pronounced by oxidative and metabolic stress or inflammatory conditions. Healthy mitochondria from the donor cell (left) move to a cancer cell (right) with damaged mitochondria. Mitochondria from cancer cells are possibly transported in opposite directions via TNTs. Mitochondrial transport in TNTs requires molecular motors that move the organelles along tubulin or actin filaments. It cannot be excluded that filaments are continuous from the donor cell into the TNT. **(B)** In the donor cell, mitochondria have to be transported towards the cell periphery into the base of TNTs. Peripheral localization of mitochondria is governed by kinesin or myosin motor proteins associated with microtubules or actin filaments respectively, tethered by Miro proteins. **(C)** Inside TNTs, healthy mitochondria are transported presumably along microtubules by kinesin motor with the assistance of TRAK adaptor and Miro, or along actin filaments by myosin with the assistance of Miro proteins. Damaged mitochondria could be possibly moved in the opposite direction by dynein with the assistance of Miro protein. **(D)** In the acceptor cell, imported mitochondria need to “switch the tracks” to be transported further within the cell. After import, mitochondria can establish functional connections with the nucleus (by means of nucleus-associated mitochondrial structures, NAMS), endoplasmic reticulum (ER-mitochondria encounter structures, ERMCS) and other organelles. The figure was created utilizing elements from Servier Medical Art (Creative Common Attribution 3.0 Generic License, https://smart.servier.com/).

Besides microtubule-associated motor proteins, Miro ensures direct interaction also with the actin motor protein myosin XIX that is hypothesized to be responsible for the movement of mitochondria *via* TNTs along actin filaments (Oeding et al., 2018; Qin et al., 2021). The same authors also mentioned the possibility of mitochondrial “docking” with myosin V or VI during their transition from the cytoplasm to TNTs. It would be interesting to find out whether this docking function of Miro proteins could be proved not only in the case of actin transport but also in the case of tubulin transport. It should be noted that mitochondria ought to “leave” TNTs on the side of the acceptor cells at their “+” termini and associate with tubulin fibers at their “+” termini. Mitochondria then move towards the “−” termini of cytoplasmic microtubules toward the perinuclear space, being propelled by dynein (see Figure 1). Here mitochondria can establish functional connection with recipient cellular organelles like the nucleus, ER or peroxisomes (Sugiura et al., 2017; Castro et al., 2018; Okumoto et al., 2018; Modi et al., 2019; Desai et al., 2020). Miro is involved not only in the “positioning” of mitochondria within recipient cells, but also in establishing functional connection of these organelles (Castro et al., 2018; Castro and Schrader, 2018; Okumoto et al., 2018; Modi et al., 2019; White et al., 2020; Guillen-Samander et al., 2021). Mitochondria from cancer cells could be possibly transported in opposite directions *via* TNTs (Caicedo et al., 2015; Lu et al., 2017). Exact regulation of mitochondrial movement directionality *via* Miro as well as the inner cytoskeletal organization of TNTs (e.g., orientation of microtubules) remains an intriguing topic for further investigation. Concerning damaged mitochondria, it is known that Miro plays an important role in the process of mitophagy, i.e., selective degradation of damaged mitochondria (Shlevkov et al., 2016; Lopez-Domenech et al., 2018; Safiulina et al., 2019a; Safiulina et al., 2019b). Moreover, Miro-assisted pulling of mitochondria along microtubules was recently described as a crucial step in the formation of mitochondrial-derived vesicles important for their quality control (Konig et al., 2021). Although much less common, intercellular transfer of damaged mitochondria has been demonstrated (Davis et al., 2014; Mahrouf-Yorgov et al., 2017; Wang et al., 2018; Crewe et al., 2021). In one case, Miro expression in recipient MSCs correlated with damage induced in donor cells by hydrogen peroxide (Mahrouf-Yorgov et al., 2017).

Additionally Miro1 and partially Miro2 have been shown to be important for mitochondrial transport towards the cell periphery, especially in case of energy-demanding processes such as migration and invasion of cancer cells (Caino et al., 2016; Lopez-Domenech et al., 2018; Wang et al., 2021). Knock-out of these two proteins results in perinuclear localization of mitochondria, with more prominent phenotype in case of Miro1/Miro2 double knock-out (Kittler, 2015). It can be expected that successful displacement of mitochondria at the periphery of the donor cell is a necessary step for the process of mitochondrial transfer *via* TNTs; and, Miro proteins play an essential role in this process. As mentioned above, Miro proteins have also been shown to be part of the MICOS complex that connects outer mitochondrial membrane and inner mitochondrial membrane, whereby maintaining the integrity of cristae structure of mitochondria during their transfer (Modi et al., 2019).

## The role of Miro proteins in cell migration and cancer metastasis

Recent findings show that localization of mitochondria within the cell is important for eventual cellular responses in the tumor niche (Senft and Ronai, 2016; Altieri, 2017; Alshaabi et al., 2020; Furnish and Caino, 2020). Constant reprogramming of cellular metabolism in order to meet the energetic demands of cancer cells requires a flexible system of mitochondrial trafficking. Distribution/positioning of functional mitochondria in cells can have an impact on energy-consuming processes such as proliferation, cell plasticity, migration and the subsequent metastatic capacity of cancer cells (Desai et al., 2013; Zhao et al., 2013; Caino et al., 2015; Rivadeneira et al., 2015; Furnish and Caino, 2020). During the process of migration and invasion of tumor cells, mitochondria are present in the cortical cytoskeleton and the whole mitochondrial network is positioned in the direction of the movement of the cells (Desai et al., 2013).

Morphological changes, changes of the cell shape and adhesion to extracellular matrix, together with the physical forces occurring during cell migration, are generated by F-actin cytoskeleton (Gardel et al., 2010). These processes are coordinated by a number of signaling events and biochemical reactions that have high demand for energy, which is provided by mitochondria. Similarly, the leading edge of migrating cells needs a supply of ATP that is produced by oxidative phosphorylation rather than by glycolysis, which is supported by re-distribution of mitochondria toward the leading edge regulated by AMP-activated protein kinase (Cunniff et al., 2016). The importance of ATP production at the leading edge of migrating cells was manifested in tumor cells with deficient oxidative phosphorylation (ρ0 cells lacking mtDNA) or in cells treated with inhibitors of mitochondrial respiratory complexes I-III and V (Caino et al., 2015).

Transport of mitochondria within cells occurs by means of their movement along actin and tubulin cytoskeleton with essential function of Miro proteins in this process. Several papers reported that inhibition of actin using specific inhibitors did not affect migration of the cells or focal adhesion formation, but inhibition of tubulin using nocodazol had a considerable effect on these processes, pointing to tubulin transport of mitochondria as playing an important role in positioning of mitochondria at the leading edge of migrating cells (Nguyen et al., 2018; Alshaabi et al., 2021). It was reported in several papers that knock-out of Miro1 results in perinuclear localization of mitochondria and in restricted mitochondrial network, but did not affect mitochondrial bioenergetics, most probably due to the compensatory mechanism of Miro2 as both proteins were shown to regulate mitochondrial morphology and cristae architecture (Nguyen et al., 2014; Cunniff et al., 2016; Schuler et al., 2017; Lopez-Domenech et al., 2018; Alshaabi et al., 2021).

An interesting work of Schuler et al. (2017) reported that intracellular position of mitochondria determines subcellular energy gradients with the highest ATP/ADP gradient in the perinuclear space that gradually declines towards the cell periphery and from the ventral to dorsal surface of the cell. These energy gradients correlate with the number and distribution of mitochondria. In Miro1^−/−^ mouse embryonic fibroblasts (MEFs), this phenomenon was highly pronounced due to restricted mitochondrial localization. Knock-out of Miro1 led to increased level of ADP and reduced ATP/ADP ratio with the highest ratio in the area of highest mitochondrial density around the nucleus, rapidly declining towards the cell periphery. This change of intracellular energy distribution in Miro1^−/−^ MEFs resulted in decreased leading edge protrusion and membrane ruffling, impaired focal adhesion formation and conclusively in reduced velocity of migrating cells (Schuler et al., 2017). Additionally, mitochondrial localization and density correlates not only with the ATP/ADP ratio but also with the level of hydrogen peroxide affecting local cellular responses to elevated mitochondrial ROS (Alshaabi et al., 2021). Consistent with this, it is known that the level of hydrogen peroxide is increased in the leading edge of migrating tumor cells (Cameron et al., 2015).

MEF cells lacking Miro1 have significantly reduced levels of peripheral hydrogen peroxide, which did not increase any further after inhibition of mitochondrial respiratory complex I with rotenone (Alshaabi et al., 2021). On the other hand, rotenone treatment induced higher level of nuclear hydrogen peroxide that was associated with elevated DNA damage response and correlated with perinuclear localization of the mitochondria in these cells (Alshaabi et al., 2021). These findings not only document that ATP and hydrogen peroxide (or ROS in general) are not simply diffused in the cell but form gradients copying the subcellular position of mitochondria, affecting the importance of the architecture of mitochondrial network and their distribution towards the “site of need” during highly energy demanding processes such as cellular migration. The importance of mitochondrial motility as an active part of the process of cell migration was demonstrated also in the model of atherosclerotic vascular disease in vascular smooth muscle cells (VSMCs) (Nguyen et al., 2018). Migration of VSMCs is regulated by mitochondrial Ca^2+^ calmodulin-dependent kinase II (mtCaMKII), the key regulator of the mitochondrial Ca^2+^ uniporter (MCU). Miro1 was found to be required for mtCaMKII-mediated mitochondrial translocation and Miro1 KO abolished migration of VSMCs cells (Nguyen et al., 2018).

Although the findings mentioned above were mostly acquired using non-cancerous cells (MEFs, VSMCs), several other papers implied the role of Miro proteins in the process of cell migration of cancer cells and their subsequent invasion and metastasis (Jiang et al., 2012; Desai et al., 2013; Li Q. et al., 2015; Caino et al., 2015; Caino et al., 2016; Mills et al., 2016; Qu et al., 2019; Wang et al., 2019; Furnish and Caino, 2020; Ghosh et al., 2022). Both proteins are expressed ubiquitously in various tissues and are not cancer-specific. *RHOT1* gene expression can be found upregulated (pancreatic adenocarcinoma, cholangio carcinoma, esophageal carcinoma, glioblastoma or acute myeloid leukemia and thymoma) as well as downregulated (adrenocortical carcinoma, ovarian serous cystadenocarcinoma, skin cutaneous melanoma). On the other hand, *RHOT2* is overall downregulated in cancer compared to normal tissue with exceptions being pancreatic adenocarcinoma, cholangio carcinoma and thymoma (as for Miro1) (Tang et al., 2017). However, relevant functional studies are missing. Several studies have shown a link between increased expression of Miro1 and Miro2 and cell migration or metastasis (Li Q. et al., 2015; Caino et al., 2016; Furnish et al., 2022)*.* Expression of Miro1 was significantly higher in pancreatic ductal epithelial cells of pancreatic cancer from patients comparing to pre-cancerous tissues, and knock-down of Miro1 in SW1990 pancreatic adenocarcinoma cell line resulted in inhibition of cell migration (Li Q. et al., 2015). Interestingly, SMAD4 protein, a tumor-suppressor involved in TGFβ signaling, has been shown to be negatively regulated by Miro1 expression (Li Q. et al., 2015). Similarly, Miro2 expression was found to be significantly higher in several types of tumors compared to normal tissue and was upregulated in metastatic cancer cells when compared to primary tumors (Caino et al., 2016; Furnish et al., 2022). Downregulation of Miro1 expression was found to regulate negatively tumor cell invasion in syntaphilin knock-down cancer cells (Caino et al., 2016). Syntaphilin is a negative regulator of tumor cell migration and invasion, and the protein is connected with the tubulin transport of vesicles and was formerly thought to play a role exclusively in neurons; however, recent research revealed that it may be functional in other cell types (Caino et al., 2016; Caino et al., 2017; Seo et al., 2018; Hwang et al., 2019; Furnish and Caino, 2020; Chen et al., 2021). Although Miro2 did not have an effect on tumor cell invasion in the model of syntaphilin knock-down cells, it did effect, together with Miro1, mitochondrial trafficking and invasion of cancer cells in the model of stress induced tumor cell invasion, indicating a mechanism independent of the syntaphilin protein (Caino et al., 2016; Lopez-Domenech et al., 2018).

## Discussion and conclusion

Miro proteins are important regulators of mitochondrial dynamics emanating their function beyond acting as mere adaptors that help movement of mitochondria along actin and tubulin fibers. Involvement in regulation of mitochondrial trafficking, regulation of mitophagy, an important tool of quality control of mitochondria, communication with other organelles such as peroxisomes, the endoplasmic reticulum or the nucleus, support of integrity of cristae structures as part of the MICOS complex, and regulation of calcium signaling that controls positioning of mitochondria within the cell, plus modulation of signaling pathways, makes the Miro proteins highly important players of regulation of mitochondrial metabolism and subsequent cellular responses (Yang M. et al., 2020; Eberhardt et al., 2020; Nahacka et al., 2021; Zinsmaier, 2021). In this review, we support the notion that mitochondria are indispensable for tumorigenesis and tumor progression (Porporato et al., 2018; Boukalova et al., 2020; Furnish and Caino, 2020). From *in vitro* and *in vivo* data we know that intercellular mitochondrial transfer could be found in tumors (Osswald et al., 2015; Tan et al., 2015; Moschoi et al., 2016; Dong et al., 2017; Bajzikova et al., 2019). Horizontal transfer of mitochondria between cells is a process that is still not well characterized, especially in cancer where detailed information is missing. The role of Miro in the process of intercellular mitochondrial transfer is supported in other disease models, where TNTs are involved (Kay et al., 2018; Kruppa et al., 2018; Panchal and Tiwari, 2021). Overexpression of Miro1 resulted in enhancement of horizontal transfer of mitochondria and improved cell recovery after ischemic or oxidative damage in different cell types (Ahmad et al., 2014; Babenko et al., 2018; Li et al., 2020; Tseng et al., 2021). Another paper showed that higher expression of Miro1 in iPSC-MSCs was responsible for superior efficacy in mitochondrial transfer compared to horizontal transfer of mitochondria observed in MSCs isolated from bone marrow in the model of cardiomyopathy (Zhang et al., 2016). This research points out to a rather general mechanism behind the regulation of mitochondrial “exchange” in different disease models, where Miro1 plays an important role in recovery of different types of cells after oxidative damage. Even though expression of both proteins Miro1 and Miro2 was found elevated in different types of patient tumors, crucial *in vitro* and *in vivo* data showing direct involvement of these proteins in the process of horizontal mitochondrial transfer in cancer are still missing. Available data we have are of an indirect character *via* involvement of Miro in the mitochondrial transport in TNTs (Qin et al., 2021; Raghavan et al., 2021).

As Miro2 is more redundant in the presence of Miro1 in terms of mitochondrial transport along tubulin and since it is not able to compensate for the loss of Miro1, it is highly probable that Miro2 would not be crucial for the process of horizontal transfer *via* TNTs and till this date Miro2 has not been documented to be involved in intercellular transfer of mitochondria. An example implying the possible role of Miro2 in horizontal transfer could be found in inter-mitochondrial communication in the heart, where Miro2 facilitates “mitochondrial nanotunneling” (Cao et al., 2019). Horizontal transfer of mitochondria is a complex process and even though Miro2 may not be a crucial player in the transfer of mitochondria per se, it could be important for the reconstruction of the mitochondrial network and re-establishing proper signalization (between mitochondria themselves or between ER and mitochondria) and finally respiration in the recipient cells. Since calcium signaling is fundamental for the mitochondrial function and both Miro1 and Miro2 have been found to equally contribute to calcium-regulated signaling (except for the mitochondrial trafficking), it is highly probable that the two proteins are equally involved in the process of re-establishing of mitochondrial function in the recipient cells.

As mentioned, TNTs are considered the most frequent means of intercellular mitochondrial transfer, but observation of their formation is limited to *in vitro* experiments. For this reason, further investigation of the formation and function of TNTs requires multimodal approaches. An example is the use of spatially defined co-cultures using microfluidic devices allowing quantitative assessment of TNTs formation as exemplified by the work of Yang et al. (2016). Ultrastructure analysis of TNTs, optimally using sophisticated microscopic methods such as FIB-SEM microscopy or CLEM, will be essential for the correct interpretation of structural details of TNTs as shown recently, providing surprising description of the TNT complex, involving smaller TNTs bundled together such that they appear as a single TNT (Sartori-Rupp et al., 2019).

We showed in our previous experimental work that mitochondrial transfer is necessary for tumor cell proliferation due to the role of mitochondria in *de novo* pyrimidine synthesis (Bajzikova et al., 2019). An interesting idea was proposed that cancer cells switch between the proliferative state to highly invasive and metastatic status by redistribution of mitochondria from the perinuclear space to the cortical cytoskeleton and the cell’s leading edge (Caino et al., 2017; Furnish and Caino, 2020). Recent data showing physical connections of the nucleus and mitochondria support this idea (Desai et al., 2020). Therefore, Miro proteins could play an important role in both mitochondrial transfer and cell invasion/metastasis. Redistribution of energetically active mitochondria to the cortical cytoskeleton was shown to be an important step for cancer cells to migrate and invade distant tissues. Even though the majority of data point to tubulin transport and Miro1 protein as crucial in the process of migration of cancer cells, as shown in the model of migration induced by PI3 kinase inhibitors, the involvement of actin transport and Miro2 remains questionable (Caino et al., 2016). More data are needed to elucidate the role of these two proteins in the process of migration and metastasis.

In this review, we discuss the notion that mitochondria and Miro proteins are essential in the two important phases of tumorigenesis. In cancer cells with aberrant oxidative phosphorylation, mitochondrial transfer is necessary to initiate tumor progression and, positioning of mitochondria within the cancer cell is crucial for further invasion and metastasis. This makes mitochondria an attractive target for translational medicine as already shown for chemotherapy of leukemia and radiotherapy of glioblastoma, where mitochondrial transfer to cancer cells from the stroma was documented to occur in the course of the treatment, possibly contributing to tumor resistance (Osswald et al., 2015; Moschoi et al., 2016).
